# History of a Case of Exophthalmos or Protruded Eye-ball

**Published:** 1830

**Authors:** Daniel Drake

**Affiliations:** Cincinnati


ART.—VII. *History of a case of Exophthalmos or Protruded Eye ball.* By DANIEL DRAKE, M. D.

As the standard works on the Surgery of the Eye, have but a limited circulation in the Western States, I shall preface the following history with a few

PRACTICAL OBSERVATIONS.

Several causes may give rise to a protrusion, more or less considerable, of the globe of the eye, the organ remaining healthy and of its natural size.

1. Accumulations of adipose matter.
2. Collections of entravasated blood, from a punctured wound extending deeply into the socket, or from blows.
3. Abscesses, from purulent inflammation in the orbit, behind the eye ball.
4. Induration and thickening of the cellular substance from inflammation.
5. Hydatids.
6. Aneurism of the ophthalmic artery ; and aneurism by anastomosis.
7. Exostoses from the bones of the socket.
8. Enlargements or hydatids of the lachrymal gland.
9. Scirrhous, cartilaginous or fungous tumours.
10. Encysted tumours, seated in the adipose substance of the orbit.

On each of these affections, I shall make a few diagnostic and therapeutic remarks.

1. When the protrusion is caused by accumulations of adipose matter, the axis of the eye remains unchanged, and vision unimpaired ; the patient experiences little or no pain, and the eye is without inflammation, until its pressure against the lids becomes such as to excite irritation. The disease often affects one eye only.

If the patient be of a full habit and inclined to obesity, general blood letting, purging and a vegetable diet, will be necessary. Mr. Ware (Posthumous Observations p. 295,) observes :

"The repeated application of leeches, on the temple and forehead, has been found of great use in subduing this morbid tendency. In one case, that came under my own care, the projection was speedily diminished by opening the temporal artery ; and, after the hæmorrhage had ceased, by converting the orifice into an issue, the discharge from which became soon very considerable. In another case, in which the protrusion occasioned great pain, and nearly destroyed vision, a perfect cure was accomplished by the application of a large caustic behind the ear. The discharge which it occasioned, when the eschar separated, was profuse ; and it was kept up nearly a month, by the insertion of a dozen peas daily."

Dr. Delafield thinks this malady in general of a scrophulous nature.

2. When displacement of the eye ball follows, speedily on a wound carried into the socket, there can be no mistake as to the diagnosis. The deep seated parts become engorged by capillary congestion. and extravasation into the cellular tissue and the cavity of the wound.

A temporary exophthalmos is the necessary consequence and will be made to disappear, by promoting the escape of coagula from the wound and causing an abatement of the inflammation, by appropriate antiphlogistic measures.

3. Phlegmonic inflammation behind the eye ball, may originate from external causes, or be, in the language of the profession, spontaneous, like the abscesses which form in other parts of the adipose membrane. It may be either acute or chronic. Mr. Ware remarks :

"If the suppuration be quick in its progress, and be not situated deep, the fluctuation of the matter may be easily felt, and the propriety of discharging it be determined on at once ; but if, as I have occasionally found, the suppuration be slow, and the matter lie considerably below the surface, the eye will be protruded before any fluctuation can be discovered ; and the existence of the matter will only be learned by paying attention to the accompanying symptoms, such as a quick pulse, white tongue, shiverings, &c." 292.

In his Operative Surgery of the Eye, Mr. Guthrie, in reference to this affection, observes—

"Sometimes, when the swelling is the result of acute inflammation, the abscess is formed with great pain; and when measures are not taken early for the discharge of the matter, its undue retention may give rise to very serious consequences; but it finds its way in general to the surface of one or other of the lids, and its evacuation, either spontaneously or artificially, leads to a subsidence of the symptoms, as well as a return of the eyeball to its natural situation, if it should have been protruded from the orbit by the size of the tumour. The pain, in these cases, is deep-seated and distressing, always accompanied by fever, and occasions considerable alarm from the swelling of the parts and the commencing protrusion of the eyeball. The upper lid swells, becomes red, œdematous, and immoveable; the conjunctiva inflames, and a viscid watery discharge takes place, which requires to be frequently removed. The eyeball is but little affected, otherwise than by its displacement from the increasing size of the abscess, which at last points and bursts. If the formation of matter cannot be prevented, the free discharge of it should be assisted by keeping the external orifice freely open, until the sac of the abscess has gradually contracted and closed. Where sinuses form, leading into the orbit, the orifices should be dilated, and their internal surfaces induced to take on a healthy action by stimulant applications in the form of injections, or by the insertion of substances which may exercise a gentle pressure upon them, such as prepared sponge, catgut, &c. If the abscess should have formed in the cellular texture and parts surrounding and supporting the eyeball, the cure will be effected without any deformity; but if it should be connected with the periosteum lining the orbit, it will frequently be found to give rise to very troublesome consequences, and often to deformity, by causing an aversiou of the lid affected by the disease. A case of this kind I have already alluded to, when on the subject of ectropium." 156-7.

From the relation which the socket of the eye and its periosteum bear to the brain and its membranes, every case of acute inflammation which may arise in the former should be met by a prompt and vigorous antiphlogistic treatment, such as general blood letting, purging, abstinence cupping, and opening a branch of the temporal artery or the angular vein. By these means the disease may be limited to the orbit, and suppuration either prevented, or the extent of the abscess much circumscribed.

4. When chronic inflammation has given rise to induration and thickening of the cellular substance of the orbit, the measures adapted to the reduction of the remedies of the inflammation; followed by such as will excite the action of the absorbent system, should be adopted. Mr. Guthrie says, it may in general be removed by alteratives, by an issue near the part, by attention to the state of the bowels, and by good air and exercise.

5. In Mr. Travers' Synopsis, p. 242, we have the following paragraph on hydatids in the socket—

"In the last annual report of the London Infirmary for diseases of the eye, is the notice of a singular case of hydatid cyst protruding the globe, with the following remark: "One of these cases was a protrusion of the eye from the orbit, by a cyst containing hydatids deeply seated in the cavity. The hydatids were evacuated by a puncture in the cyst; the eye returned into its natural situation, and the patient was completely cured. This is the only instance of such an affection that has occurred since the opening of the Infirmary."

6. The aneurismal tumours which protrude the eye ball, are either of the ophthalmic artery, or of small branches, constituting aneurism by anastomosis. This is a most painful affection. Mr. Travers and Mr. Dalrymple have each reported a case of aneurism by anastomosis. Guthrie, p. 168, says,

"The first sensations experienced by the patients of commencing disease were a sudden snap in the orbit, followed by pain, and accompanied by a whizzing noise in the head. In a few hours, a copious effusion of a limpid fluid took place into the eyelids, the pain increased in severity, so as to become intolerable. The eye soon began to protrude, and a tumour became perceptible, in both cases evidently of an aneurismal character. The pain was now accompanied by a noise in the head, which one patient compared to the blowing of a pair of bellows, the other to the rippling of water, and both the pain and noise were insupportable, when the head fell below the natural level."

Mr. Guthrie adds, *loco sitato.*

"I have seen one case of true aneurism of the ophthalmic artery, of both sides, which terminated fatally. The symptoms were similar to those above mentioned, but no tumour could be perceived; the eye was gradually protruded until it seemed to be exterior to the orbit, but vision was scarcely affected. The hissing noise in the head could be distinctly heard, and was attributed to aneurism. On the death of the patient, an aneurism of the ophthalmic artery was discovered on each side, of about the size of a large nut; the vena ophthalmica cerebralis was greatly enlarged, and obstructed near where it passes through the foramen lacerum orbitale superius, in consequence of a great increase of size the four recti muscles had attained, accompanied by an almost cartilaginous hardness, which had been as much concerned in the protrusion of the eye as the enlargement of the vessels."

The remedy for this dangerous affection, is tying the carotid artery of the affected side. In the case reported by Mr. Dalrymple, it was perfectly successful ; but in the one previously treated by Mr. Travers, the progress of the cure was not quite so favourable, till the patient, five months after the operation, suffered abortion with profuse hæmorrhage, when the tumour suddenly disappeared. In the case of Mr. Guthrie's patient with true aneurism of the ophthalmic arteries, no operation was performed, as the disease existed on both sides.

7. Exostosis of the bones of the orbit is a rare disease. In its protrusion the eye ball necessarily changes the direction of its axis. If deep seated the character of this tumour cannot be ascertained without cutting down to it. Demours, (*Traite des Mal. des Yeux tom.* 1.) has recorded a case in which Brassant effected a cure by the application of caustic, which produced a partial exfoliation, after which the eye returned to its place. In the opinion of Mr. Guthric, if an operation be resorted to, a chissel or saw offers the best chance of success, but should be used with caution.

8. The lachrymal gland does not appear to be very liable to disease, though so often affected by mental emotions. In nearly twelve thousand patients Mr. Guthrie has met with but one case of suppuration, and none of scirrhus of this gland The latter malady has, however, been repeatedly met with, by other practitioners, and he gives the following, as the history of its origin and progress.

" The symptoms of scirrhus are at first obscure ; a slight uneasiness is felt at the outer part of, and within the upper edge of the orbit, which is sometimes attended by a sudden discharge of tears from the eye, more particularly when this uneasiness amounts to pain, and is increased by paroxysms : flashes of light are subsequently perceived in the eye, and about the same time the enlargement of the gland is observed. This is soon followed by displacement of the eye inwards, with more or less of direct protrusion. As the gland enlarges it becomes irregular and knotty to the touch, the eyelid is stretched over it, the conjunctiva falls into a state of low chronic inflammation, and the lower eyelid is everted. The pain is, in this stage of the disease, very severe and lancinating, principally at night. The eye continues to protrude until it appears to rest upon the cheek. In some cases vision is totally destroyed, in others it is only impaired, in a third set the patient sees objects double, and in one instance I saw the eye greatly protruded without any defect of sight. The changes in the structure of the eye may be scarcely observable, or the reverse. 170.

Extirpation is of course the only remedy, and this has been several times performed with success.

9. Cartilaginous and sarcomatous tumours are occasionally met with. They may lie beneath the periosteum and implicate the bone, when the case is serious, if not hopeless. Mr. Travers (*Synopsis*, p. 243) observes—

"I have seen several cases of this description where the tumor appeared to extend the depth of the orbit, and was presenting on the nasal side. Their anterior edge is thin, being bound down to the orbitar circumference, but when they protrude and compress the globe to blindness, as is sometimes the case, it is to be inferred that they have attained considerable bulk posterior to the globe. I once removed one on the abductor side of the globe, by scraping it clean away from the bone ; it was of the hardness of cartilage and of great extent. I am unable to say whether the disease returned, having soon afterwards lost sight of the patient. The impression I had of the case was unfavourable, from the character, as well as the extent and connections of the tumor.

10. Encysted tumours in the socket are more common than any others. They may be compared to wens in other parts of the adipose system. Scarpa has favoured the profession with a short, but perspicuous and satisfactory paper on these morbid structures, from which I shall make such extracts as seem necessary to illustrate their history.

"A soft tumor contained in a membranous capsule is sometimes formed in the cellular adipose substance which surrounds and insinuates itself between the muscles of the eye, and the other parts contained in the orbit, similar in every respect to the *encysted* tumors, which are generated in the other cellular parts of the body. In the greater number of cases, the tumor is as large as a pigeon's egg, and sometimes even larger. In general it contains a compact fatty substance ; occasionally it is divided internally into two compartments in one of which is found a mixture of a liquid and a cretaceous substance, and in the other a glutinous fluid resembling the white of egg ; and in some cases the tumor is wholly filled with a limpid or sanious fluid.

The origin or root of the *encysted* tumor is generally seated below the eyeball more or less deeply in the cavity of the orbit ; but it very rarely arises from the bottom of this cavity, and enlarges so as to press the eyeball beyond the orbit and palpebræ without its becoming perceptible. Most frequently, as I have stated, it originates below the globe of the eye, or a little to the side of it, and as it increases in volume, it projects from the orbit against the lower eyelid, so as to elevate it in the form of tumor, and press it downwards upon the cheek sometimes to the extent of half an inch.

During its enlargement, the tumor of necessity has a constant tendency to force the eyeball out of its natural situation ; and as its origin is below the inferior hemisphere of the eyeball, this organ is gradually pushed upwards towards the superior eyelid and out of the orbit : so that, finally, the pupil of the displaced eye no longer corresponds, either in its position or direction, to that of the sound side. But if the *encysted* tumor, increasing from beneath the eye, incline more towards the nose than the temple, the eye is forced outwards and forwards towards the external angle of the palpebræ, and *vice versa.* In this unnatural position of the eyeball, notwithstanding its immobility, and the great distension under such circumstances to which the optic nerve is subjected, the power of sight in the displaced eye is not in all instances of this kind entirely abolished.

The deformity arising from this disease is frightful ; and it is easy, from the circumstances enumerated, to foresee, how various and unpleasant must be the consequences attendant on it, as double vision, the continual discharge of tears upon the cheek, frequent pain in the eye and head, repeated ophthalmia, and the painful impression of light. 441.

In reference to the diagnosis of this and other tumors of the orbit, Demours remarks—

"It is difficult to decide in the beginning of the exophthalmos whether the displacement results from exostosis, an encysted tumour, or a fungous excrescence. It is only by the event that we can know in certain cases the nature of the cause of the protrusion. Nevertheless we may presume that exostosis is present, if the patient be syphilitic, that the tumour is scrophulous if the signs of a constitution struma are present, or that an encysted tumour exists if wens have appeared on other parts of the body." *Traite tom* 1. p. 486.

For the removal of these tumours, Scarpa gives the following instructions.

"There is undoubtedly no other really efficacious means of remedying this disease than the extirpation of the *encysted* tumor from the cavity of the orbit, which, being effected, it is not afterwards difficult, as experience has proved, to restore the eyeball to its natural situation.

This operation is executed in the following manner. The patient being placed horizontally, with his head a little raised, and firmly held by an assistant, the surgeon, with the fore and middle fingers of one hand, should draw the skin of the eyelid tense upon the tumor, and with a convex edged bistoury divide the skin of the eyelid and orbicular muscle transversely in the direction of the fibres of that muscle and the lower arch of the orbit. This incision ought to be made with the hand unsupported, in order that the cyst of the tumor may not be included in it; and should also extend a little towards the two angles of the eye, according to the size of the tumor, in order to render the other steps of the operation within the orbit easy and expeditious; observing carefully in making this incision to avoid the lachrymal duct at the internal angle. On the protrusion of the cyst from the wound, the surgeon should separate it carefully from the lips of the wound, and to as great a depth as possible within the orbit, and then introduce on one side of it a fine hook with a single or double point, by which, having gained a secure hold of the body of the tumor, he may draw it gently towards him. In this position, with the point of the bistoury, or with the points of scissors adapted to it, he should separate it around from all its other adhesions within the orbit, as well as from its principal and deeper attachments to this cavity. It will rarely happen that, in separating the upper part of the cyst from the lower eyelid, the portion of conjunctiva, which unites the eyelid to the lower hemisphere of the eyeball, will be injured; as, in the course of the disease, this expansion of the conjunctiva, following the protrusion of the eyeball and palpebræ out of the orbit, is, as it were, everted, and consequently sufficiently removed from the summit of the subjacent *encysted* tumor, to prevent its being included in the dissection of it, and the separation of the surrounding parts. On dividing the deepor roots of the tumor, it is sometimes found that they are, contrary to all expectation, hard and coriaceous; on this account, after the removal of the tumor, it is proper to introduce the point of the finger gently to the bottom of the cavity from which it has been removed, in order to ascertain whether any portions of this hard substance remain; in which case it will be necessary to remove them by means of the small hook and point of the scissors. If the cyst, on being first laid hold of by the hook, should accidently burst, and the whole of the serous, albuminous, or puriform fluid contained in it should be in consequence discharged from it, since, as I before stated, the tumor sometimes contains only a fluid inclosed in one or more membranous compartments, it is not necessary on this account to relinquish the principal object of the operation, or that of removing the whole cyst; which may be accomplished in the manner just now laid down, although, indeed, with greater difficulty than when the tumor is firm and admits of being drawn out by degrees from the margin of the orbit.

The hæmorrhage is never considerable after this operation ; the first dressing, therefore, consists only in gently filling the cavity, from which the tumor has been removed, with lint. The inevitable consequences of the operation are, very great pain in the orbit and head, inflammation of the eyelids, and sometimes of the face and neck ; for the removal of which symptoms, recourse must be had to bleeding, according to the patient's strength, antiphlogistic purgatives, emollient and anodyne topics, and low diet. If, on the fifth day from the operation, suppuration has commenced, the dressing is to be removed. In some cases it is necessary to do this sooner ; when, for instance, there are certain indications that the intensity and continuance of the pain in the orbit and head arise from grumous blood collected in the cavity, before occupied by the tumor, although filled with lint ; on giving a discharge to which the pain ceases.

As soon as the general and local symptoms are quieted, a healthy suppuration and granulation commences at the bottom of the wound, and the cavity containing the tumor gradually diminishes, and finally heals. During the treatment, the surgeon should be careful to keep the external lips of the wound in the eyelid separated, by the introduction of a folded dosil, in order to give a free issue to the matter discharged from the orbit, and to prevent the edges of the wound from uniting before the cavity, situated in the soft parts of the orbit in which the tumor was lodged, has closed. The cure is effected in general in four or five weeks.

The eyeball, notwithstanding the removal of the extraneous body by which it had been forced out of its natural position, does not return so soon to its proper situation as might perhaps be imagined by those who are unacquainted with the subject. The long continued retraction of the elevator muscle of the eyeball, and the elongation of the depressor, when the globe of the eye is pressed upwards and outwards by the tumor, or, in a similar manner, the shortening of the abductor and relaxation of the adductor muscle, when the eye has been forced out of the socket towards the temple, are the principal and obvious causes by which the complete cure of the disease is retarded. Immediately after the operation, indeed, when the eyeball is gently pressed on in the direction contrary to that in which it was forced out of its place, it is easily restored to its situation ; but on the pressure being removed, it returns to its former vitiated position. As soon, therefore, as the general and local symptoms, arising from the operation, have abated, and the eyeball on being pressed back can be contained and covered by the eyelids, it will be proper, by pressure upon it, to direct it towards its natural situation, and carefully maintain it there, by means of graduated compresses and a suitable bandage. We have some examples in which, even without this aid, after a very considerable time, however, the muscles of the eye have spontaneously recovered their tone and reciprocity of action ; but it is undoubtedly a great advantage, confirmed by practice, to be able to obtain it more promptly and without much inconvenience to the patient, and not to leave the work entirely to nature." 444—446.

In addition, I shall transcribe from Mr. Guthrie's more recent work the following practical observations.

"It must be first ascertained whether the tumour can be removed without injuring the eye, and that point being decided in the affirmative, the operation is to be done in that way which will give the greatest facility to its execution, and cause the least subsequent deformity. If it tend towards the external canthus, and has displaced the eye inwards, the outer commissure should be divided, and room obtained in this way to raise the lid, so as to allow it to be dissected out. If the tumour should be, on the contrary, towards the inner angle of the eye, that part should not be enlarged on account of the lachrymal apparatus, and the operation must be done by cutting through the eyelid, in the direction of the line of the orbit, which method will succeed when the tumour is small; or by fairly dividing the eyelid in a perpendicular direction through the tarsal cartilage, by which each part may be turned outwards, and the eyeball and the anterior part of the tumour be fully exposed. Dr. Monteath relates the history of a case in which he performed the operation in this way, on account of an encysted organized tumour, of the size of a plum. The eyelid reunited perfectly, and regained nearly its natural power and extent of motion. When the first incision has been made in either of these ways, the tumour should be laid hold of with the hook, and carefully dissected out with the scissors, or small knife; and if any portions of it should remain, which may be ascertained by examination with the finger, they should be carefully removed by the hook and scissors. The after-treatment must depend on circumstances: if the tumour has been completely removed, the healing process may be allowed to go on as speedily as possible; but if any portion of the cyst or sac should have been allowed either designedly or accidentally to remain, the external opening should be kept in direct communication with it, until the wound fills up from the bottom.

When the tumour is posterior to the eyeball, or connected with it, it can scarcely ever be removed, unless the eye be extirpated at the same time; and, as its nature can seldom be ascertained previously to the operation, the removal of the whole will in general be the most advisable proceeding; this step will also be the less regretted, as the eye will, in most instances, be either useless or disorganized."
160—162.

With these extracts, which I trust will not be without interest to many of the readers of the Western Journal, I shall proceed to report a recent case of exophthalmos from a tumour of the last species, with an account of the operation for its removal.

### Case.

Miss E. H. aged 17 years, was the subject of the following case. She is of feeble frame and constitution, but not sickly, except that for some years she has been afflicted with head ache. In April 1829, a small quantity of lime fell into her left eye, but the next day it was quite well. A month afterwards it was discovered by her friends, that the eye ball was either enlarged, or projected from its socket a little. The protrusion gradually increased, but not at a regular rate, being sometimes stationary for weeks, and then advancing, for a time, with greater rapidity. When she was feverish or had severe attacks of head ache, the protrusion was always observed to be greatest. She had no pain in the eye or its socket, nor was the organ inflamed. At first her sight was unimpaired ; but it gradually decayed, and by the month of October was quite extinguished.

Near the end of the ensuing May, she was brought to Cincinnati to be placed under my care. At this time, the protrusion was so great, as to occasion a most unpleasant deformity. The eye lids, still, however, by an act of volition could be brought over the eye ball ; and in sleep they nearly covered it ; but to effect this, the upper lid had become so attenuated, that the colour of the iris and pupil could be seen through it. At times a spasmodic action of the oblique muscles, projected the globe so far out of the socket, that the lids contracted behind it and gave her pain, and the same state could be produced by pressure with the finger.

The projection was directly forward, and the pupil preserved its parallelism with the other. The two eyes moved in perfect harmony, and no pain was excited in the affected organ or its orbit by any of its movements. The pupils, also, acted in concert, and in every degree of light, exhibited the same diameter. When the light, however, was excluded from the sound eye, the pupil of the other instantly expanded—showing that the motions of the iris of the unsound eye were not governed by the impress of light upon that membrane, but upon the optic nerve of the other organ.*

On feeling with a finger round the anterior part of the socket, a firm, smooth tumour could be felt beneath the orbitar arch, extending from one canthus to the other. It bore pressure without pain, and did not recede. At first view, the globe of the eye appeared to be enlarged, but on measuring the two with *callipers*, this was found not to be the case. The cause of the exophthalmos was now quite obvious ; but what might be the nature of the tumour, and whether it could be removed without extirpating the eye, remained to be ascertained.

Uncertain whether it was encysted or sarcomatous, and unable to decide whether it could be extracted without the removal of the organ, I determined to commence the operation in such manner as would provide for any state of the parts that might be found to exist. The patient having been previously dieted was placed, as for cataract. In imitation of Richerand, as quoted by Demours, I divided the external commissure of the eye lids, and then cut through the duplicature of the conjunctiva, from near the inner canthus, to the incision at the outer, so as to liberate the upper lid. A little dissection enabled me to feel the rounded edge of the tumour, which occupied the front of the entire concave of the upper part of the

* It is well known, that in amaurosis of one eye, the pupil is generally dilated, even under the brightest light. Now a question arises, why in this case the incidence of light upon the sound eye does not cause the pupil of the other to contract, as in the example before us ? It would seem, that the integrity of the sensibility of the optic nerve of the amaurotic eye, is necessary to a sympathy of its iris with the retina of the other. In Miss H.'s eye, the nerve was probably healthy, but, as we shall presently see, compressed by a tumour, which arrested the impression made upon its extremity, but did not destroy its capacity for sympathizing with the retina of the other side. If this view be correct, may we not conclude, that in the cases in which the pupil of an amaurotic eye continues associated in its movements with the other, the blindness is owing to mechanical pressure on the affected nerve, and not to the destruction of its sensibilities? And are we not thus furnished with one means of diagnosis ? I make the suggestion in a note, as too vague and uncertain, to merit a place in the report of the case.

socket, and felt like a compressed cyst. Unlike many other tumours of the same species, it did not advance on being cut down upon, nor could it be made to recede before the finger. With a pair of curved scissors, I separated its adhesions with the socket, to a considerable depth, when hooking it with a tenaculum, I endeavoured to bring it forward; but it was found to adhere firmly at or towards the bottom of the socket, and the pulling gave pain. On applying greater force to the hook, it tore out, and there escaped with the blood, a considerable quantity of gelatinous fluid, part of which was transparent, and the rest semi-opake and more indurated. By this discharge, the tumour was sensibly diminished towards the outer canthus, but retained its figure towards the inner, where I then inserted the tenaculum, and again resorted to the scissors, carrying them as deep into the socket as could be done without injuring the optic nerve, at its entrance through the foramen. On again applying force to the hook, it tore out, and a fresh discharge of the contents occurred. A finger could now be introduced into the cyst, and carried nearly to the bottom of the orbit. Employing it as a director, I siezed different parts of the sack and tore them off, but its posterior attachments remained, and pulling at them gave pain as before. With a finger and the handle of a scalpel I removed an additional quantity of the semi fluid contents. The cord of nerves and muscles could now be distinctly felt, and the globe of the eye, which retained all its movements, could be pressed into the socket, but no return of sight was the consequence. In this state of things, the question arose, whether I should finish the operation by the extirpation of the eye and the entire contents of the orbit, or leave the remnants of the sack to slough away, or shrink up and remain subject to absorption in the adipose substance of the socket. As the patient was a young female, it seemed desirable, if possible, to retain the sclerotic ball, that she might be supplied with an artificial eye, having movements in harmony with the other, and I determined on finishing the operation as it then was. For the purpose of removing any portions of the coagulated contents of the sack which might remain, I threw into the cavity a small syringe full of tepid water. This measure I now think was unnecessary, and had the disadvantage of injecting the cellular substance beneath the conjuctiva of the lower eye lid as it issued from the wound, producing an œdema which interfered by its magnitude, with the dressing of the eye. The incision in the external commissure was drawn together with slips of adhesive plaster, the upper eyelid brought over the eye ball as far as it could be made to cover it, and a compress and roller applied.

The hæmorrhage attending the operation was inconsiderable; and the pain, although not trifling, was borne with great equanimity. The patient's pulse being weak, she was directed to take 50 drops of laudanum, and be put to bed with her head raised.

Two hours after the operation she was affected with nausea and head ache; and, her pulse being somewhat tense, she was ordered to lose 20 ounces of blood, which afforded considerable relief. At night she lost, at a second bleeding, eight ounces: and had the compresses wet with tepid water. In the course of the night she vomited, and had a sinapism applied to the epigastrium. She slept but little. Next day on removing the compress, the chemosis was very great, and the eye more protruded than before the operation. All compression was now removed from it, and a slice of fresh beef applied.*

It is unnecessary to give a diary of the case from this time forward. Fever, head ache, insomnolency, general œdema of the head and face, and great tumefaction and pain about the affected eye, supervened, and continued for several days; during which I had the aid of Doctors Atlee, Marshall and Warren. In our anxiety to save the brain from inflammation, she lost, with that previously taken, about 60 ounces of blood, none of which except the last was sizy. Cathartics, low diet,

* The soothing and antiphlogistic effect of fresh meat, from an animal just killed, when applied to the eye after severe injuries, is well known to all, who retain a recollection of the *materia chirurgica* of the GOUGERS, who formerly tore out each others eyes for the amusement of the good people of the Western country.

subtepid affusions to the head, a blister to the neck, and calomel until it affected her mouth, were, also, resorted to, and employed to such an extent, as to induce an irritable state, accompanied with head ache increased by rising up, which required and was signally allayed by the preparations of morphia. A favourable suppuration at length ensued, and the eye was dressed with elm poultices. A gelatino-purulent discharge took place from the socket, and the incision was kept open for some time with dossils of lint. The dressings were carefully examined, but no portions of the cyst were at any time seen to come away.

It was soon observed, that the cornea would slough, but it was not finally detached for a month. When this took place, the capsule of the vitrous humour was lacerated, to promote the escape of that fluid and diminish the size of the sclerotic tubercle, so as to promote its return into the socket. Under a course of topical astringents, stimulants and mild escharotics, the chemosis slowly disappeared, and at the end of five weeks, the globe was evidently receding, while her general health was much improved. A fold of the thickened conjuctiva however, almost cartilaginous and nearly insensible to escharoties, projected between the ball and the lower eye lid, at once preventing the return of the former, and producing an ectropion of the latter. This was at length removed with a pair of scissors, and the depressed and almost paralyzed lid brought up, when it was found that it met the upper lid. A dry compress and bandage were now employed, and in a week the lid was able to retain its place and perform its motions.

At this time, twelve weeks after the operation, the eye is free from inflammation, pain, and discharge of any kind ; the lower lid has its former convexity, but the upper is somewhat flatted from the eye projecting less than it ought. The lids meet each other except when she makes an effort to open them, but do not sink in as when the eye has been extirpated. She bears pressure well over every part of the socket, no tumour can be felt any where, nor does any thing indicate a reproduction of the disease. The sclerotic ball moves in concert with the sound eye, and seems well fitted to receive an artificial cornea.

I have thought this case, at least as well worth reporting, as many others, which find their way into the journals ; and indulge the hope, that when taken in connexion with the systematized and practical view of Exophthalmos, with which it is introduced, it will not be without an ordinary interest to the profession in the West, if not elsewhere. I have been the more desirous of publishing it, because at one time, and, still, in some degree, I was disposed to regard my decision (during the operation) to preserve the eye instead of extirpating it as wrong. The inflammation was probably greater, than if extirpation had been practised ; and for several weeks, there seemed little probability, that the organ would ever resume its natural situation. As it is, the patient is evidently better off than if the eye had been removed.

Tumours in the orbit of the eye are so rare (at least in the Western States) that I have never before met with a case ; and have seen but one example of *fungus hæmatodes* of the eye ball. It was in a child 3 or 4 years old. On extirpating the contents of the orbit, I found the whole a mass of morbid structure, with many spiculæ of bone, some of which had such contiguity to the periosteum, as materially to interfere with the movements of the scalpel. The socket filled with granulations in a promising way ; but when they reached the cut edges of the conjunctiva, cicatrization did not take place, and a fungoid tumour soon began to project from between the lids. The child was taken back to the country where it died.

Cincinnati, August, 1830.

